# O.4.4-3 Developing a bespoke undergraduate physical activity course for a Portuguese Medical School - the VANGUARD European Union Erasmus+ project

**DOI:** 10.1093/eurpub/ckad133.200

**Published:** 2023-09-11

**Authors:** Romeu Mendes, Ana Barbosa, Alberto Caldas Afonso, Idalina Beirão, Henrique Barros

**Affiliations:** EPIUnit - Instituto de Saúde Pública, Universidade do Porto, Porto, Portugal; Laboratório para a Investigação Integrativa e Translacional em Saúde Populacional (ITR), Porto, Portugal; Administração Regional de Saúde do Norte, Porto, Portugal; EPIUnit - Instituto de Saúde Pública, Universidade do Porto, Porto, Portugal; Laboratório para a Investigação Integrativa e Translacional em Saúde Populacional (ITR), Porto, Portugal; EPIUnit - Instituto de Saúde Pública, Universidade do Porto, Porto, Portugal; Laboratório para a Investigação Integrativa e Translacional em Saúde Populacional (ITR), Porto, Portugal; Instituto de Ciências Biomédicas Abel Salazar, Universidade do Porto, Porto, Portugal; Centro Hospitalar Universitário de Santo António, Porto, Portugal; Laboratório para a Investigação Integrativa e Translacional em Saúde Populacional (ITR), Porto, Portugal; Instituto de Ciências Biomédicas Abel Salazar, Universidade do Porto, Porto, Portugal; Centro Hospitalar Universitário de Santo António, Porto, Portugal; Unit for Multidisciplinary Research in Biomedicine (UMIB), Universidade do Porto, Porto, Portugal; romeuduartemendes@gmail.com; EPIUnit - Instituto de Saúde Pública, Universidade do Porto, Porto, Portugal; Laboratório para a Investigação Integrativa e Translacional em Saúde Populacional (ITR), Porto, Portugal

## Abstract

**Purpose:**

Embedding physical activity in the undergraduate curricula of frontline health professionals is one of the policies recommended by the World Health Organization and the European Commission to promote health-enhancing physical activity (HEPA).

The VANGUARD project is supported by the European Union Erasmus+ Programme, and aims to embed physical activity in the undergraduate curricula of healthcare professionals in European countries.

Within this project, this work aimed to describe the bespoke development of a physical activity and health course integrated into the undergraduate medical curriculum of a Portuguese Medical School.

**Project or policy description:**

The School of Medicine and Biomedical Sciences of the University of Porto (ICBAS) was the invited Medical School to implement the VANGUARD project in Portugal - led by the Institute of Public Health of the University of Porto (ISPUP).

An auscultation process with the undergraduate medical students was held to evaluate the importance of HEPA in their curriculum and assess the preferred operational characteristics of including a physical activity and health course in the Integrated Master’s degree in Medicine.

A course proposal structure was then developed by the VANGUARD project Portuguese team from ISPUP, the Director of the Integrated Master’s degree in Medicine, and the Coordinator of the Medical Education Office of ICBAS, based on the students’ acceptability, the Medical School constraints, and VANGUARD project goals and resources.

The course integrates 26 lessons, from physical activity technical terminology, epidemiology and guidelines, going through its relation with noncommunicable diseases (using #MovementForMovement resources), to Portuguese National Health Service brief intervention tools and community-based exercise programs - in a total of 20 hours of contact, including continuous evaluation and final case studies analysis. The course will be offered on an optional basis to 2nd-year medical students, on an online training platform, using a self-paced learning method.

After embedding this course in ICBAS, it will be presented to the Council of Portuguese Medical Schools to be scaled up nationally.

**Conclusions:**

Medical doctors and Medical Schools are key stakeholders in HEPA promotion, especially in the health sector. Successful implementation of HEPA policies requires tailored solutions to organizations and direct involvement of target groups in policy development.

